# Regional Influence of Radiation Oncology Residency Training on Job Securement over Two Time Periods

**DOI:** 10.7759/cureus.4495

**Published:** 2019-04-18

**Authors:** Abhinav V Reddy, Daniel W Golden, Jeffrey V Brower, Stanley L Liauw

**Affiliations:** 1 Radiation Oncology, Johns Hopkins Medicine, Baltimore, USA; 2 Radiation Oncology, The University of Chicago Medicine, Chicago, USA; 3 Radiation Oncology, University of Wisconsin, Madison, USA

**Keywords:** radiation oncology residency, regional influence, job market, job saturation, job securement, portability

## Abstract

Purpose

Recent reports have noted conflicting predictions regarding the future of the radiation oncology job market. Residents and practicing radiation oncologists (ROs) report perceptions of an increasingly saturated market. An important factor contributing to the job landscape is the potential geographic maldistribution of ROs in the United States. Given the importance of the evolving job market and appropriate supply and demand for future ROs, this study investigated whether residency training region influences employment region and whether “portability” of residency training has changed over time from 2003-2015.

Methods

Radiation oncology residency graduates were identified from Association of Residents in Radiation Oncology (ARRO) directories from 2003-2012. This information was cross-referenced with the American Society of Radiation Oncology directory to determine current employment location. The region of residency training and employment were categorized into four regions per the US Census Bureau: Northeast (NE), South (S), Midwest (MW), and West (W). The change in “portability” of residency training over time was determined from the results of an anonymous internet-based survey which provided information on year of graduation and location of first job. “Portability” was defined as the rate at which a trainee in one region could find employment in another region. From the survey, two cohorts were identified: early (graduated from 2003-2006) and late (graduated from 2012-2015).

Results

Current employment location was available for 817/1168 (70%) residents identified in the ARRO directories from 2003-2012. The percentages of residents who trained in the NE, S, MW, and W were 29%, 28%, 27%, and 15%, respectively. The percentages of residents with current employment in the NE, S, MW, and W were 20%, 34%, 22%, and 24%, respectively. Residents were more likely to remain employed in the region in which they trained (p < 0.05), with 58% having current employment in the region of their training. Residency graduation year and location of first job (in the United States) were available for 139/198 (70%) survey respondents. Portability of residency training did not significantly change from 2003-2012 with 49% of the early cohort securing their first job in the region in which they trained compared to 57% of the late cohort (p = 0.39).

Conclusions

This study suggests that recent residents are not moving to different geographic regions at an increased rate than previous and that residents are more likely to find employment in the region in which they trained.

## Introduction

The ability to secure employment upon graduation from radiation oncology (RO) residency is thought to have become more difficult as both residents and current radiation oncologists (ROs) feel that the market is more saturated than previous years [1-5]. Although an analysis in 2010 projected that there may be shortage of ROs with the demand exceeding the supply from 2010-2020 [6], a more recent study predicted that the supply of ROs may outpace the demand from 2015-2025 [7]. A separate issue is the potential geographic maldistribution of ROs in the United States [8, 9]. Aneja et al. reported a greater concentration of practicing ROs in regions with higher education levels, higher incomes, and lower unemployment rates [8].

Given the implications of this topic and the recent expansion of RO residency programs, investigation of trends within the RO job landscape is justifiable. Although others have shown that RO residents tend to find employment in the same region of their training, it is unclear whether this trend has changed over time [10]. This is the first study to report on “portability” of RO residency training over time. This study hypothesized that residency training location influences employment location and that residency training portability has increased over time from 2003-2015.

Part of this study was presented at the 2018 American Society for Radiation Oncology Annual Meeting (Poster: Reddy AV, Brower JV, Golden DW, Liauw SL. Impact of Radiation Oncology Training Program Location on Region of Job Securement across Two Time Periods. American Society for Radiation Oncology; October 23, 2018).

## Materials and methods

Residency graduates were identified from archived Association of Residents in Radiation Oncology (ARRO) directories from 2003-2012, which included the names of all trainees in each residency program. This information was cross-referenced with the American Society for Radiation Oncology directory to determine location of current employment. The location of residency training and employment were categorized in four regions: Northeast (NE), South (S), Midwest (MW), and West (W) per the US Census Bureau [11]. Graduation year and location of first job were available for a subset of respondents who completed an internet-based survey as described in another study [4]. From the survey, two cohorts were identified, an early cohort which graduated from 2003-2006 and a late cohort which graduated from 2012-2015. Portability was defined as the rate at which a trainee in one region could find employment in another region. All statistical analyses were performed with JMP version 13.0 software (SAS Institute, Cary, NC). Chi-square test was used to test differences between categorical variables.

## Results

A total of 1168 residents were identified in ARRO directories, of which 817 (70%) had known employment location in the United States. By region, 29%, 28%, 27%, and 15% of residents were trained in the NE, S, MW, and W regions, respectively. Analysis of employment location demonstrated that 20%, 34%, 22%, and 24% were employed in the NE, S, MW, and W regions, respectively (Table 1). Overall, graduating residents were more likely to be employed in the South (34%) with the next most common location being the West (24%).

**Table 1:** Training and employment information for radiation oncology residency graduates from 2003-2015.

Variable	n or %
Total residents	1168
Total residents with current employment in the United States	817
Median # of residents in program	7
Mean # of residents in program	7.8
Region of Residency Training	
Northeast	238/817, 29%
South	232/817, 28%
Midwest	222/817, 27%
West	125/817, 15%
Region of Current Job	
Northeast	164/817, 20%
South	278/817, 34%
Midwest	182/817, 22%
West	193/817, 24%
Current job in same region as residency training	472/817, 58%

**Figure 1:**
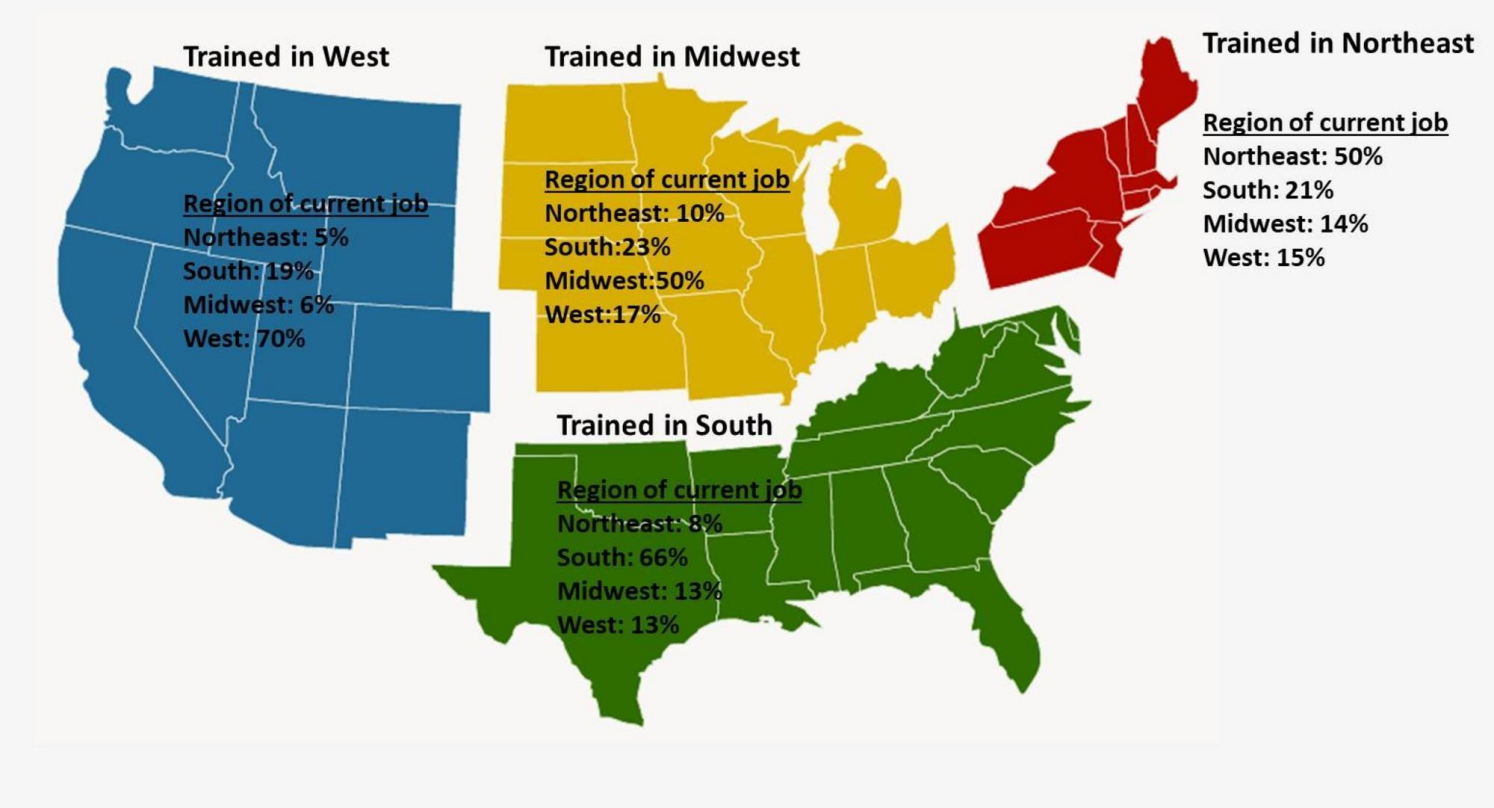
Map of radiation oncology training region and current employment region.

Graduating residents were more likely to remain employed in the region of their residency training, with 58% having current employment in the same region as their training (p < 0.001, chi-square). When compared to trainees from the Northeast and Midwest, those from the South and West were more likely to be employed in their respective regions. Seventy percent of residents from West programs and 66% of residents from South programs were employed in their respective regions versus 50% of residents from Northeast and Midwest programs (Figure 1).

The ability of trainees to find employment in the same region as their residency training did not change over a 13-year period (2003-2015). Of the 198 survey respondents, 142 (71%) had information available for both graduation year and location of first job. Of these 142 ROs, 139 graduated from 2003-2006 (n = 47) or from 2012-2015 (n = 92). Upon analysis, portability of training was not significantly associated with year of residency graduation. Of the early cohort, 49% had their first job in their respective residency training region compared to 57% of the late cohort (p = 0.39, chi-square) (Table 2). When analyzed by state, 30% of the early cohort found their first job in the same state as their residency training versus 33% of the late cohort (p = 0.70, chi-square) (Table 2).

**Table 2:** Portability of radiation oncology residency training over two time periods.

Graduation year	First job in same region as residency training	First job in same state as residency training
2003-2006	49% (23/47)	30% (14/47)
2012-2015	57% (52/92)	33% (30/92)

## Discussion

This study suggests that RO residents are not moving to different US Census Bureau-based regions at increased rate over a 13-year period. Additionally, there is a regional bias with regards to employment, with the majority (58%) of residents being employed in the same region as their residency training. This agrees with findings from a prior analysis by Ahmed et al. [10], which demonstrated that among RO residents graduating in 2013, 62% were employed in the same region as their residency training. This regional bias is likely multifactorial involving a combination of personal factors and opportunities between residency programs with local hospitals/institutions. Notably, survey studies [4, 12] have revealed that “geographic location” was a factor deemed very important by ROs in selecting their residency program and post-graduate employment.

A point of contention is whether residents’ dissatisfaction with the current job landscape is due to an oversupply of ROs, a maldistribution of ROs, or a combination of both. Chowdhary et al. [13] shed light on this topic. The authors demonstrated that the Northeast trains far more residents than jobs available in that region with roughly five trainees for every four jobs (proportion of total jobs to graduates, 0.78). The analysis reported here within supports this conclusion as only 50% of residents trained in the Northeast were employed there, which is significantly lower than the rate of trainees in the South (66%) and West (70%) who stayed in their respective regions for employment. Chowdhary et al. also demonstrated that the Midwest had more jobs available than the number of graduating residents, with roughly two trainees for every three jobs (proportion of total jobs to graduates, 1.54) [13]. This study’s findings suggest that Midwest-trained graduates are just as likely to take these jobs as graduates from other regions. This differs from employment in the South and West where jobs in these two regions are predominantly taken by graduates who trained there. This mismatch is consistent with the geographic maldistribution of ROs described by Aneja et al. who showed that ROs are more concentrated in urban and metropolitan areas in coastal regions as opposed to rural areas in the Midwest [8].

One potential consequence of a saturated job market may be the need to practice in a different region after completion of training. However, this study suggests that the proportion of trainees finding employment outside of their training region or state has not changed from 2003-2015, with similar ratios in the early and late cohorts. While this metric of portability of training is proposed to serve as practical information that may help assess the job market and the degree of saturation, it is an imperfect surrogate as it excludes many complex factors that can undoubtedly influence both residency and job selection (e.g., spousal or family needs, the desire to practice in a metropolitan vs. rural setting, the desire to prioritize job “fit” over geography, etc.). Another limitation of this metric is the lack of granularity of the data. The US Census Bureau regions and even individual states include large areas and diverse communities. This metric of residency training portability would be imperfect for the resident who trained in a “desirable” West Coast city but secured employment in an “undesirable” location in the same region or state. Despite the limitations, the findings provide valuable information regarding the evolving RO job landscape.

## Conclusions

In conclusion, this report suggests that RO residency graduates from 2012-2015 are not moving to different geographic regions at an increased rate than those from 2003-2006. Radiation oncology residency graduates are also more likely to settle into practice within the region in which they trained.
